# Sorafenib Improves Survival in Metastatic Hepatocellular Carcinoma: A Case Report

**DOI:** 10.4021/wjon240w

**Published:** 2010-11-02

**Authors:** Filipe Nery, Luis Graca, Manuel Ribeiro, Henrique Guimaraes, Helena Pessegueiro Miranda

**Affiliations:** a Internal Medicine Department, Centro Hospitalar do Porto – Hospital Santo Antonio, Portugal; b Surgical Department, Centro Hospitalar do Porto – Hospital Santo Antonio, Portugal; c Radiology Department, Centro Hospitalar do Porto – Hospital Santo Antonio, Portugal

**Keywords:** Hepatocellular carcinoma, Metastatic disease, Sorafenib

## Abstract

Hepatocellular carcinoma (HCC) is a very common cancer. Curative treatments and local ones are well validated. Sorafenib, a multi-kinase receptor inhibitor was introduced in 2007 for advanced HCC in patients with preserved liver function. HCC is known to be resistant to systemic chemotherapy, and there are no validated therapies improving survival for metastatic disease. Herein, we report a case of a 45 years old woman with chronic hepatitis B infection submitted to a right hepatectomy in May 2001 for an hepatic tumor with more than 10 cm wide, confirmed as a HCC moderately differentiated. Three years later, a solitary pulmonary metastasis was documented and a metastectomy was done. In February 2009, the patient started on sorafenib 400 mg twice daily due to an inferior mediastinal metastasis with a vena cava thrombus associated. Computed tomography (CT) scan done 13 months after revealed a consistently mass reduction in more than 50% and a clinically well patient without important collateral effects. HCC is a highly vascularized tumor and sorafenib is known to inhibit both tumor angiogenesis and tumor cell survival. It is already approved for the treatment of advanced and metastatic renal cell cancer. In our case, the combination of two well done surgical procedures and the posterior use of sorafenib when a metastasis was found in an inaccessible surgical place with macroscopic vascular invasion, led to a long survival without important side effects.

## Introduction

Liver cancer is the sixth most common cancer all over the world, which is consistently the third most common cause of cancer death in recent years, with a very heterogeneous distribution worldwide depending on the etiology of the subjacent chronic liver disease [1, 2]. In Portugal, the incidence of hepatocellular carcinoma (HCC) is rising, with a mortality rate that affects a quarter of HCC hospital admission [3].

Clinical, radiologic, biochemical and cytological diagnostic HCC criteria are well recognized and reproducible, and treatment modalities vary according to tumor dimension, number of nodules, localization, liver function and subjacent cirrhosis, disseminated disease, and local resources. Surgical treatment has a curative aim, varying from tumor resection with free margins to liver transplantation. In MELD (Model for End-Stage Liver Disease) era, for allocating patients in a waiting liver transplantation list, a change was necessarily made to those with HCC (as it may arise in patients with normal or nearly-normal liver function with a low MELD score putting them in a low position for transplantation) [4, 5]. Depending on the centers, Milan criteria or more extended ones as San Francisco are used to incorporate patients in a waiting list for transplantation according to survival expectancy [6, 7].

Local HCC treatments exist as neo-adjuvant or down-staging options, including trans-arterial chemo-embolisation, radio-frequency ablation, percutaneous ethanol injection and trans-arterial radioembolisation, with the aim of reducing tumor dimension and serving as a bridge to surgical resection or transplantation [8].

Increased expression of multidrug resistance transporters and active intracellular metabolism in HCC results in an intrinsic resistance to chemotherapy drugs, conducing systemic chemotherapy as to be historically ineffective [9-11].

Successive stages of liver damage, from fibrosis to cirrhosis and HCC, depend on formation of new vessels and the establishment of an abnormal angioarchitecture. It seems that platelet-derived growth factor (PDGF), transforming growth factor-b1 (TGF-b1), fibroblast growth factor (FGF) and vascular endothelial growth factor (VEGF) play a potent pro-fibrogenic and pro-angiogenic role in liver disease [12]. It was based on this relationship between angiogenesis and HCC that sorafenib, an oral multi-targeted tyrosine kinase inhibitor (of Raf-1, VEGFR2 and 3, PDGFR-β, FLT3 and KIT), was considered as a promising therapy and approved in 2007 for the treatment of advanced HCC. By competing with adenosine triphosphate (ATP) for the ATP-binding site of the catalytic domain of the tyrosine kinase, sorafenib prevents the intracellular signaling which leads to angiogenesis [13, 14].

Sorafenib was sequentially involved in phase I, II and III studies [15-17]. A survival advantage of nearly 3 months was proved in a phase III study that compared patients with advanced HCC under sorafenib versus placebo, confirmed by a more recent meta-analysis, and changing the way how to treat these patients [17, 18].

Recently, 15 cases of complete remission attributed to sorafenib treatment in HCC with extrahepatic spread were reported in Japan though it is rarely reported in other countries, even because there is no clear indication on the use of sorafenib in this kind of patients [19].

We report a clinical case of a patient with chronic hepatitis B infection with metastatic HCC disease controlled with sorafenib treatment.

## Case Report

In May 2001, a 45-year-old woman was referred to our center due to a liver mass. She had chronic hepatitis B, diagnosed in 1997 after routine blood test. No viral treatment was done. In May 2001, due to abdominal pain localized at right hypochondria, an abdominal ultrasound was done, which revealed a mass with 10 cm wide in the posterior segments of the right hepatic lobe, and the patient was referred to our center. Computed tomography (CT) abdominal scan confirmed a hypodense mass with peripheral contrast enhancement in arterial phase with portal washout, highly suggestible of a HCC. AgHbs, AntiHbc and AntiHbe were positive. Delta virus was negative, as was hepatitis C virus and HIV1/2. Liver analysis revealed an AST of 155 U/L, ALT of 192 U/L, PAL of 130 U/L, GGT of 170 U/L and α-fetoprotein (AFP) of 60.77 µg/L. The HBV DNA was negative. Coagulation was normal. Her ECOG performance status was 1 and Karnofsky performance status scale of 90%. As it was a unique mass in a non-cirrhotic liver (liver function was preserved and there were no signs of chronic liver disease in imagery), a right hepatectomy was performed. Histology revealed a moderately differentiated HCC with a size of 8 x 10 x 14 cm and a trabecular pattern with an immunocytochemistry revealing negativity for AFP and positivity for CAM 5.2. Remnant liver revealed a chronic hepatitis with mild fibrosis. Transaminases and AFP levels returned to normal values after surgery (3.4 µg/L). No antiviral therapy or any adjuvant systemic chemotherapy was started.

Three years later (May 2004), in a routine CT scan, a nodular lesion with 2 cm wide with tissue density in the right inferior pulmonary lobe was found. AFP levels were normal (3.9 µg/L). As it was a unique metastasis, a metastectomy was done. Histology was concordant with a HCC metastasis.

**Figure 1:**
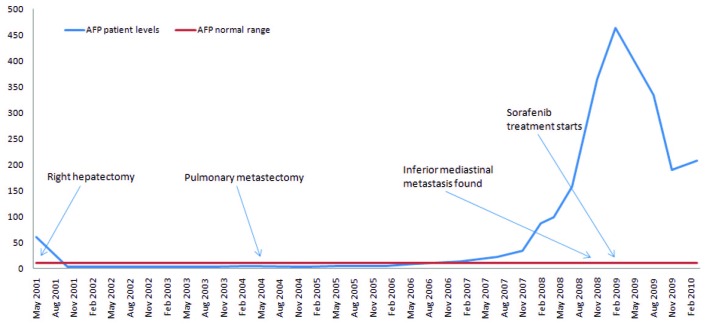
α-Fetoprotein evolution and principal events along the clinical course.

The patient remained under surveillance. In January 2007, AFP levels started slowly rising (Fig. 1). A more tightened program with regular thoracic and abdominal CT scans was started, all being negative for disseminated disease. In spite of its low sensitivity for HCC, a PET scan was done in July 2008, which was described as normal. At that time AFP was of 157.4 µg/L, transaminases were normal and HBV DNA was of 2908 IU/mL.

**Figure 2:**
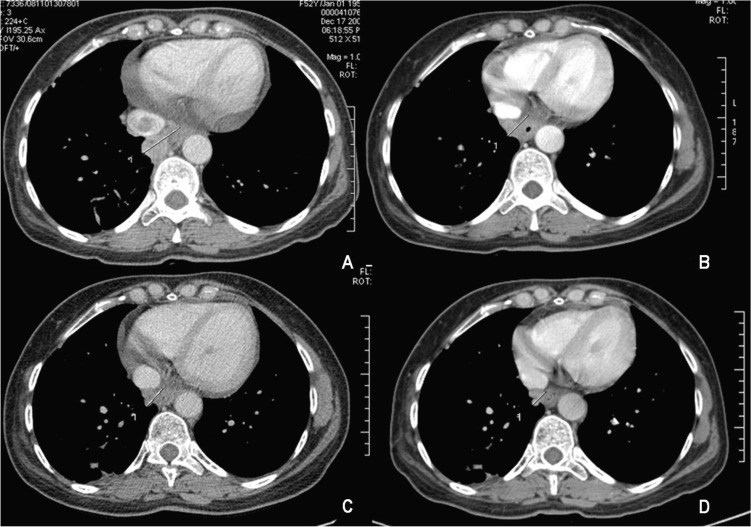
Mass involution under sorafenib treatment: (A) December 2008; (B) May 2009; (C) November 2009; (D) March 2010.

A rising AFP contrasted with the absence of demonstration of any tumor in repeatedly imaging techniques. In December 2008, AFP peaked at 365.4 µg/L. At that time, a dynamic CT identified an inferior mediastinal mass of 3.5 x 3.3 cm associated with a vena cava thrombus. Sorafenib (400 mg twice daily) was started in February 2009 (AFP of 464 µg/L, DNA VHB of 7310 UI/mL, normal transaminases) resulting in a decline of AFP values and a reduction of more than 50% of the metastatic mass (15 mm wide in March 2010) and of vena cava thrombus size (Fig. 2). Antiviral treatment with lamivudine 100 mg/day was started simultaneously resulting in an undetectable HBV DNA 3 months after. The patient is asymptomatic, reporting only transitory hair loss during treatment.

## Discussion

Our patient had a chronic hepatitis B infection, recognized in 1997 due to a disturbed hepatic profile, never treated, and a HCC that developed in a non-cirrhotic liver. HCC surveillance benefit is uncertain in this subpopulation of hepatitis B female carriers younger than 50 years old without cirrhosis [20]. In spite of that, surveillance strategies are well documented [21].

The HBV can cause HCC, directly by HBV DNA integration in the host genome and by persistent expression of viral proteins (such as HBx and LHBs: Large envelope protein) that are capable of oncogenes activation, inducing oxidative stress and genetic instability, or indirectly due to hepatocellular injury induced by chronic HBV infection and hepatocyte regeneration, conferring an annual incidence of HBV-related HCC in patients with chronic hepatitis B that ranges from 2% to 5% and with a lifetime risk for HCC approximately 10-25% [22, 23]. Our patient presented with an advanced disease, with a mass of more than 5 cm wide and cancer-related symptoms, conferring a dismal prognosis by the time of referral. Bigger tumors are less differentiated and a more common vascular and lymph invasion is expected, predisposing such patients to disseminated disease or recurrence.

As the patient had no evidence of cirrhosis and no macroscopic invasion of vascular structures, a right hepatectomy was performed as an intention to treat procedure.

By the time when the solitary pulmonary metastasis was found, metastectomy was made, as there was no evidence of liver relapse. Surgical resection of 1 or 2 solitary metastasis is recommended in the absence of intrahepatic HCC, improving survival [24].

Nowadays, AFP is not recommended for HCC surveillance, but it is recognized as a risk factor for HCC [25]. It was the rising of AFP during the follow-up that alerted the practitioner to a new probable metastasis elsewhere and made a change in the surveillance conduct. A metastasis was found in an inaccessible localization with a vena cava thrombus associated. At that time there was no place for surgical resection and systemic therapy was considered. As no systemic chemotherapy is recognized to improve survival in disseminated HCC, sorafenib was tried.

In our case, prolonged survival was related not only to a proper surgical approach (right hepatectomy and metastectomy) but also to the use of sorafenib, surpassing life expectancy at the beginning and by the time when metastatic disease was documented.

The patient is under sorafenib treatment since 17 months ago, who is clinically well, and with a documentation of more than 50% tumor reduction revealed in a CT scan 13 months after sorafenib was started.

Hepatocellular carcinoma is a highly vascularized tumor. Sorafenib in known to inhibit both tumor angiogenesis (via VEGF and PDGF signaling) and tumor cell survival (RAF kinase signaling-dependent and signaling-independent mechanisms), and is already approved for the treatment of renal cell advanced and metastatic disease [26]. In Japan, its use in metastatic HCC in patients with preserved liver function (Child-Pugh A) is consensual [19]. Very few case reports in the western world document complete remission of HCC metastatic disease (both to the lung) [27, 28].

The protracted time course of the HCC in this patient was very uncommon. Sorafenib seems a very exciting therapy for advanced HCC, and may also have very important benefits in particular patients with metastatic HCC. Phase III studies in patients with HCC and metastatic disease should come out quickly to put sorafenib (or other molecule with the same properties) in this context in the everyday clinical field.
